# Poly (ADP-ribose) Polymerase Inhibitors in Patients with Metastatic Castration-Resistant Prostate Cancer: A Meta-Analysis of Randomized Controlled Trials

**DOI:** 10.3390/medicina59122198

**Published:** 2023-12-18

**Authors:** Zheng Chao, Zefeng Wang, Le Li, Yi Jiang, Yunxing Tang, Yanan Wang, Xiaodong Hao, Chunyu Zhang, Xiangdong Guo, Weimin Yu, Fan Cheng, Zhihua Wang

**Affiliations:** 1 Department of Urology, Tongji Hospital, Tongji Medical College, Huazhong University of Science and Technology, Wuhan 430030, China; m202276257@hust.edu.cn (Z.C.);; 2 Department of Urology, Renmin Hospital of Wuhan University, Wuhan 430030, China; 3 Department of Obstetrics and Gynecology, Tongji Hospital, Tongji Medical College, Huazhong University of Science and Technology, Wuhan 430030, China; 4 Department of Interventional Radiology and Vascular Surgery, Peking University First Hospital, Beijing 100034, China

**Keywords:** prostate cancer, PARP inhibitors, survival, adverse events, meta-analysis

## Abstract

*Context*: Several recent randomized controlled trials (RCTs) have reported on the survival benefits of poly (ADP-ribose) polymerase inhibitors (PARPi) compared to standard-of-care (SOC) treatment (enzalutamide, abiraterone, or docetaxel) in patients with metastatic castration-resistant prostate cancer (mCRPC). However, there is a limited integrated analysis of high-quality evidence comparing the efficacy and safety of PARPi and SOC treatments in this context. *Objective*: This study aims to comprehensively analyze the survival benefits and adverse events associated with PARPi and SOC treatments through a head-to-head meta-analysis in mCRPC. *Evidence acquisition*: A systematic review search was conducted in PubMed, Embase, Clinical trials, and the Central Cochrane Registry in July 2023. RCTs were assessed following the Preferred Reporting Items for Systematic Reviews and Meta-analyses (PRISMA) guidelines. The systematic review was prospectively registered on PROSPERO (CRD42023441034). *Evidence synthesis*: A total of 8 studies, encompassing 2341 cases in the PARPi treatment arm and 1810 cases in the controlled arm, were included in the qualitative synthesis. The hazard ratio (HR) for radiographic progression-free survival (rPFS) and overall survival (OS) were 0.74 (95% CI, 0.61–0.90) and 0.89 (95% CI, 0.80–0.99), respectively, in the intention-to-treatment patients. For subgroup analysis, HRs for rPFS and OS in the BRCA-mutated subgroup were 0.39 (95% CI, 0.28–0.55) and 0.62 (95% CI, 0.38–0.99), while in the HRR-mutated subgroup, HR for rPFS was 0.57 (95% CI, 0.48–0.69) and for OS was 0.77 (95% CI, 0.64–0.93). The odds ratio (OR) for all grades of adverse events (AEs) and AEs with severity of at least grade 3 were 3.86 (95% CI, 2.53–5.90) and 2.30 (95% CI, 1.63–3.26), respectively. *Conclusions*: PARP inhibitors demonstrate greater effectiveness than SOC treatments in HRR/BRCA-positive patients with mCRPC. Further research is required to explore ways to reduce adverse event rates and investigate the efficacy of HRR/BRCA-negative patients.

## 1. Introduction

A significant proportion of patients inevitably progress to a state of metastatic castration resistance, which represents the terminal clinical phase in the intricate trajectory of prostate cancer evolution. In such cases, the standard-of-care (SOC) treatment options commonly employed include chemotherapy, with docetaxel or cabazitaxel, and second-generation antihormonal therapy, encompassing abiraterone or enzalutamide [1,2]. However, the meager survival benefits provided by these current therapeutic strategies have spurred an intensified exploration for alternative or combinatorial drugs, ranging from radiotherapy to immunotherapy and microbiota-targeted therapy [1,3,4]. Nevertheless, it is disheartening to note that these endeavors have not succeeded in significantly improving overall survival outcomes for patients with mCRPC [1,3,5].

Recently, the success of PARP inhibitors in other solid tumors has sparked interest in their potential application in mCRPC treatment, leading to multiple RCTs comparing PARP inhibitors with SOC treatment [6,7,8,9]. As mutations of homologous recombination repair (HRR) genes (e.g., BRCA1/2) are found in approximately one-quarter of cases of mCRPC, multiple randomized clinical trials have been conducted to explore the efficacy and safety of PARP inhibitors compared to SOC treatments [10,11,12,13,14,15,16,17,18,19,20,21,22]. Notably, due to the relatively better survival benefits observed in patients with BRCA1/2/ATM alterations in the treatment arm of the PROfound trial, olaparib has been approved and recommended for those with HRR gene alterations who have progressed to mCRPC after receiving previous next-generation hormonal drug treatment [13]. In addition, other PARP inhibitors, such as talazoparib, niraparib, veliparib, and rucaparib, have also been tested in clinical trials for mCRPC and have reported encouraging data on the therapeutic performance of PARP inhibitors in terms of survival, further demonstrating the tremendous potential and necessity to use them in treating this lethal malignancy [12,17,18,20].

Despite having similar strict inclusion criteria, these high-quality randomized controlled trials (RCTs) have displayed inconsistent results regarding survival benefits and adverse events, partly due to disparities in participant numbers, HRR gene mutation status, treatment durations, and other factors. For instance, KEYLYNK-010 reported limited survival benefits and an unfavorable side effect profile of the treatment arm with a PARP inhibitor, while TRITON3 showed advantageous radiographic progression-free survival (rPFS) over the SOC arm and comparable rates of grade 3 or higher adverse events [19,20]. Moreover, although similar improvements in rPFS were observed for patients with BRCA1/2 alterations in all RCTs, it remains controversial whether PARP inhibitors can be applied to mCRPC in patients without BRCA or even HRR gene mutations, despite recent evidence of novel mechanisms beyond inhibition of DNA repair in tumor cells [17,23].

Therefore, we performed this systematic review and meta-analysis to integrate all updated high-quality RCTs, aimed at analyzing primary endpoints and adverse events and exploring the efficacy of PARP inhibitors among specific patient subgroups. By synthesizing and integrating the available evidence, we hope that this study will provide guidance for defining the appropriate scope of PARP inhibitor application and support clinicians in making informed decisions regarding treatment options for mCRPC.

## 2. Materials and Methods

### 2.1. Search Strategy and Data Extraction

The review protocol was registered on PROSPERO (CRD42023441034) and conducted following the Preferred Reporting Items for Systematic Reviews and Meta-analyses guidelines [24]. PubMed, Embase, Clinical trials, and the Central Cochrane Registry were searched for RCTs using PARP inhibitors to treat mCRPC and published before 15 July 2023. The search keywords were as follows: (“PARP” OR “PARP inhibitors” OR “Olaparib” OR “talazoparib” OR “rucaparib” OR “niraparib” OR “veliparib”) AND (“mCRPC” OR “metastatic castration-resistant prostate cancer”). Next, two independent reviewers performed a literature screening. Of note, 4 publications of RCTs belong to the same study PROfound (NCT02987543), and two publications of RCTs belong to the same study with clinical trial number NCT01972217. We carefully compared all the publications affiliated with the same RCT and used the most updated and complete data.

### 2.2. Inclusion and Exclusion Criteria

The following studies were included: (1) randomized controlled trials; (2) patients with histologically or cytologically diagnosed mCRPC; (3) studies exploring the comparison between PARPi and SOC; (4) SOC treatment group studied was novel hormonal agents (NHA); (5) studies reported data on rPFS or OS.

The following studies were excluded: (1) single-arm trials; (2) reviews, letters, case reports, and protocols; (3) pharmacokinetics studies; (4) studies that do not provide data on relevant evaluation indicators; (5) non-English language.

### 2.3. Measure of Effect

OS and rPFS assessed by a blinded independent central review were the primary endpoints of interest. Other endpoints included in this meta-analysis were time to first subsequent therapy (TFST), time to PSA progression (TTPP), PSA response rate (a confirmed PSA decrease of at least 50%, PSA RR), and objective response rate (ORR). For safety analysis, rates of all grades, ≥3 grades, and serious treatment-emergent adverse events (AEs) were analyzed (graded according to the National Cancer Institute Common Terminology Criteria for Adverse Events version 4.03). The key results of the meta-analyses are summarized in Supplementary Table S1.

### 2.4. Risk of Bias Assessment

The risk of bias for individual nonrandomized studies was analyzed in accordance with Cochrane recommendations using RevMan 5.3 by two independent reviewers. Studies with a significant risk of bias were excluded from the quantitative synthesis.

### 2.5. Statistical Analysis

We performed a meta-analysis of the mCRPC studies. The hazard ratio (HR) was calculated to evaluate the OS, rPFS, and TFST. The odds ratio (OR) was estimated to evaluate the PSA response and ORR, as well as AEs. All estimates were expressed with their 95% confidence intervals (CIs). On this basis, we divided the patients into four subgroups according to their HRR and BRCA mutation status to discuss whether the different mutation statuses would have different outcomes in terms of efficacy benefit. All meta-analyses were performed using a random-effects model and produced into forest plots using Cochrane Collaboration ReviewManager software (RevMan 5.3). We used R 4.2.3 to conduct a sensitivity analysis by eliminating one by one method to evaluate the consistency of the results. *p* < 0.05 (two-tailed) was considered statistically significant, and I^2^ > 50% was defined as high heterogeneity.

## 3. Results

### 3.1. Study Selection

**Figure 1:**
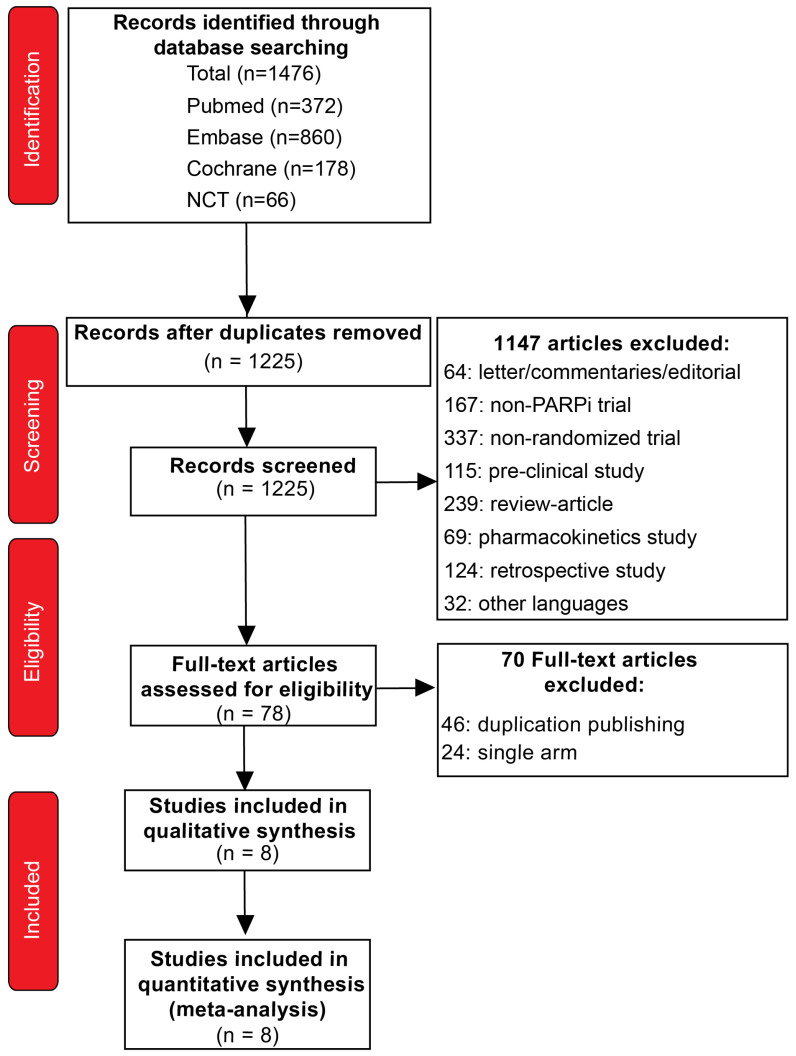
The PRISMA flow chart, detailing the article selection process.

The initial search identified 1476 publications, and a total of 1225 publications remained after the elimination of the duplicates. Then, 1147 articles were excluded after screening the titles and abstracts, and full-text reviews were performed on 78 articles. According to the selection criteria, we filtered eight studies comprising 4151 patients for inclusion in this meta-analysis. The entire process of initial screening and the reasons for excluding studies are illustrated in Figure 1. The characteristics of the included studies and patients are shown in Table 1 and Table 2. All these studies were published between 2018 and 2023, and were multicenter, prospective, large-scale clinical RCTs. Each risk of bias for the included studies was analyzed using RevMan 5.3 according to Cochrane recommendations, and the methodological quality of most studies was deemed good (Figures S1 and S2).

### 3.2. Efficacy

#### 3.2.1. rPFS

**Figure 2:**
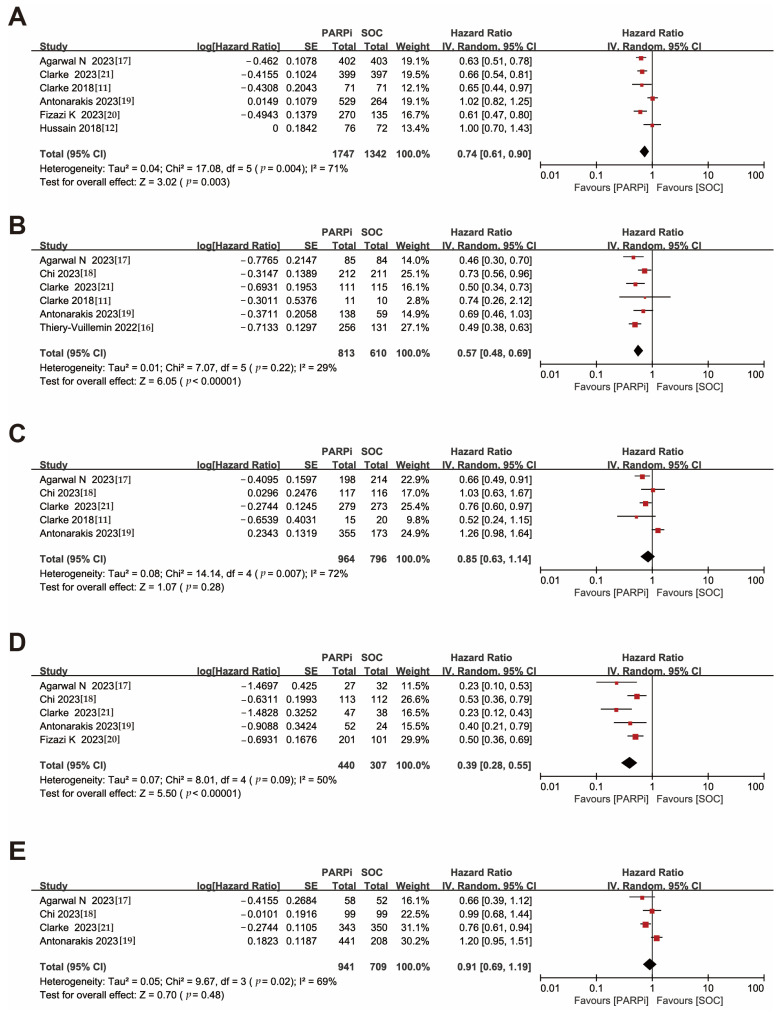
Forest plots showing the association of rPFS in mCRPC patients with or without DNA damage repair gene mutation. (**A**). Overall patients. (**B**). HRR gene-mutated patients. (**C**). Non-HRR gene-mutated patients. (**D**). BRCA gene-mutated patients. (**E**). Non-BRCA gene-mutated patients. rPFS: radiographic progression-free survival; mCRPC: metastatic castration-resistant prostate cancer; HRR: homologous recombination repair [11,12,16,17,18,19,20,21].

This meta-analysis was conducted on trials reporting rPFS as the primary endpoint and not differentiating between mutation status. The results showed that the PARPi treatment demonstrated superior efficacy (HR, 0.74; 95% CI, 0.61–0.90) compared to the control treatment (Figure 2A). Sensitivity analyses were performed, and the results showed good concordance between the trials (Figure S3A).

A similar favorable result for PARPi was obtained among patients with HRR gene mutations (HR, 0.57; 95% CI, 0.48–0.69), while no significant advantage of PARPi over standard therapy was demonstrated (HR, 0.85; 95% CI, 0.63–1.14) in populations without detectable HRR gene mutations (Figure 2B,C).

Notably, sensitivity analysis showed that even after excluding KEYLYNK-010, PARPi still exhibited improved efficacy in patients without HRR gene alterations (HR, 0.74; 95% CI, 0.62–0.88), underscoring the need for further exploration of PARPi efficacy in a wider range of patients (Figure S3B).

For patients with BRCA gene mutations, the PARPi treatment group showed a significant advantage (HR, 0.39; 95% CI, 0.28–0.55). In contrast, PARPi did not demonstrate therapeutic benefits for patients without BRCA gene mutations (HR, 0.91; 95% CI, 0.69–1.19) (Figure 2D,E), and the sensitivity analysis likewise showed that the KEYLYNK-010 had a large impact on the results (Figure S3C,D).

#### 3.2.2. OS

**Figure 3:**
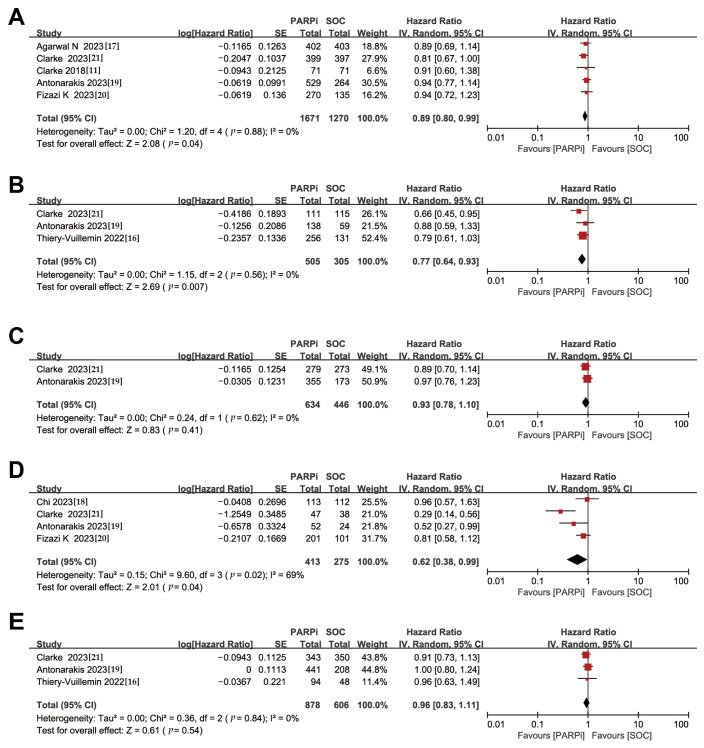
Forest plots showing the association of OS in mCRPC patients with or without DNA damage repair gene mutation. (**A**). Overall patients. (**B**). HRR gene-mutated patients. (**C**). Non-HRR gene-mutated patients. (**D**). BRCA gene-mutated patients. (**E**). Non-BRCA gene-mutated patients. OS: overall survival; mCRPC: metastatic castration-resistant prostate cancer; HRR: homologous recombination repair [11,16,17,18,19,20,21].

OS was considered a secondary outcome in the studies included in this meta-analysis. For the overall patient population, even though individual trials had negative results, a conservative model was used to derive a slight benefit for PARPi treatment compared to NHA (HR, 0.89; 95% CI, 0.80–0.99) (Figure 3A). Considering the very low heterogeneity of the original study, which became statistically significant after meta-analysis with a larger sample size (Figure S3E).

Similar to the findings for rPFS, in the subgroup of patients with HRR gene mutations, PARPi treatment demonstrated improved overall survival (HR, 0.77; 95% CI, 0.64–0.93), while no significant improvements were observed in non-HRR gene-mutated patients (HR, 0.93; 95% CI, 0.78–1.10) (Figure 3B,C).

In the BRCA gene-mutated subgroup, PARPi treatment also showed a therapeutic advantage (HR, 0.62; 95% CI, 0.38–0.99), but the evidence for this advantage in patients without BRCA gene mutations is currently inconclusive (HR, 0.96; 95% CI, 0.83–1.11) (Figure 3D,E). Nevertheless, sensitivity analyses showed heterogeneity in this finding (Figure S3F), possibly due to studies with insufficient OS maturation. This emphasizes the need for more clinical trials to follow up on whether PARPi is effective in improving overall survival in patients without BRCA gene mutations.

#### 3.2.3. Disease Progression and Relief

Based on the comprehensive information disclosed in the included studies, we evaluated four outcomes related to disease progression and relief in the intention-to-treat populations: TFST, TTPP, PSA RR, and ORR. In terms of disease progression, both TFST (HR, 0.72; 95% CI, 0.57–0.89) and TTPP (HR, 0.73; 95% CI, 0.54–0.98) were significantly reduced in the PARPi treatment arm. As for disease relief, PSA RR (OR, 1.52; 95% CI, 1.10–2.10) and ORR (OR, 1.97; 95% CI, 1.27–3.04) also favored the PARPi treatment (Figure S4A–D). Despite substantial heterogeneity among the studies, the consistent conclusions consistently favored the selection of PARPi treatment (Figure S3G–J).

### 3.3. Safety

**Figure 4:**
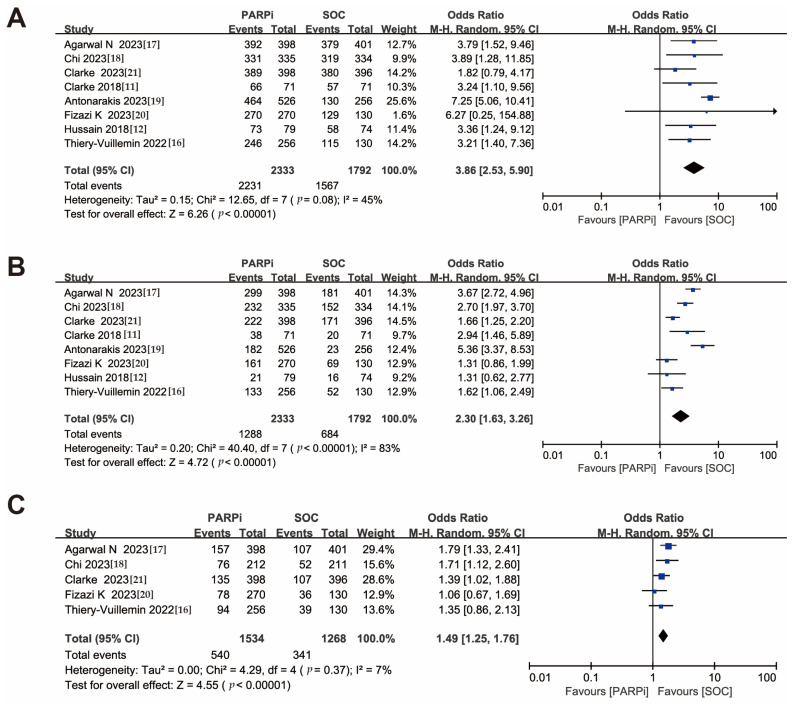
Forest plots showing the association of treatment safety in mCRPC patients. (**A**). All grades of adverse event. (**B**). Grade ≥ 3 of adverse event. (**C**). Serious adverse event. mCRPC: metastatic castration-resistant prostate cancer [11,12,16,17,18,19,20,21].

All eight included studies reported overall adverse events and grade ≥ 3 adverse events, with five studies reporting serious adverse events. The PARPi treatment group had a higher risk of all grades of adverse events (OR, 3.86; 95% CI, 2.53–5.90) compared to the SOC treatment group, as well as a higher risk of grade ≥3 adverse events (OR, 2.30; 95% CI, 1.63–3.26) and serious adverse events (OR, 1.49; 95% CI, 1.25–1.76) (Figure 4A–C). We ranked the types of side effects based on their frequencies and ultimately identified four hematologic-related side effects, hypertension, and fatigue, as indicators for further analysis. Among these, anemia and fatigue had the highest incidence rates, with odds ratios of all grades of adverse events of (OR, 6.01; 95% CI, 3.78–9.56) and (OR, 1.45; 95% CI, 1.22–1.74), and odds ratios for grade ≥3 adverse events of (OR, 9.73; 95% CI, 5.44–17.41) and (OR, 1.12; 95% CI, 0.76–1.65), respectively (Figure S5A–D). Of interest, hypertension did not significantly differ in either overall (OR, 0.76; 95% CI, 0.47–1.23) or grade ≥ 3 adverse events (OR, 0.75; 95% CI, 0.51–1.11) (Figure S5E,F).

PARPi treatment also showed a higher risk for the remaining three hematologic-related side effects: Thrombocytopenia (OR, 5.45; 95% CI, 2.61–11.40), neutropenia (OR, 3.96; 95% CI, 1.77–8.86), and leukopenia (OR, 5.28; 95% CI, 3.30–8.43) (Figure S5G,I,K). In grade ≥ 3 adverse events, thrombocytopenia (OR, 5.45; 95% CI, 2.90–10.21) and neutropenia (OR, 4.50; 95% CI, 1.18–17.16) showed similar trends (Figure S5H,J), but the association with leukopenia (OR, 5.48; 95% CI, 0.34–88.82) was not statistically significant, especially after removing the studies leading to heterogeneity (Figure S5L).

## 4. Discussion

In contrast to recent published systematic reviews [25,26], which primarily involve a constrained number of trials, often lacking randomization, potentially leading to incomplete or biased conclusions, this exhaustive meta-analysis, encompassing a corpus of eight meticulously executed prospective randomized controlled trials (RCTs), unequivocally substantiates that the application of poly (ADP-ribose) polymerase (PARP) inhibitors in the therapeutic management of metastatic castration-resistant prostate cancer (mCRPC) confers remarkable enhancements in both overall survival (OS) and radiographic progression-free survival (rPFS) across all patient cohorts. This notable improvement is observed not only among the general population but is especially pronounced in individuals exhibiting BRCA/homologous recombination repair (HRR) mutations.

BRCA1/2, the foremost DNA repair gene loci to be identified, as well as the most widely acknowledged marker for mutation testing concerning the application of PARP inhibitors, have played a pivotal role in this field [27,28]. Efforts have also been made to investigate other genes involved in homologous recombination repair (HRR) prior to the initiation of PARP inhibitor treatment, such as ATM, CDK12, CHEK2, and many others [29,30,31]. However, a consensus linking specific mutations to the application of PARP inhibitors, apart from BRCA1/2, has yet to be reached. Our findings indicate that patients who tested positive for mutations in the BRCA or HRR genes can derive therapeutic benefits from PARP inhibitor treatment, although some heterogeneity was observed in OS within the subgroup of BRCA-mutated patients. Importantly, the variability in the examination of HRR genes across different trials impairs the efficacy of conclusions establishing the subgroup of patients with HRR mutations as “potentially profited patients”.

Even in prostate cancer cases lacking HRR alterations, the combination therapy of PARP inhibitors with androgen receptor inhibitors (ARi) holds great promise due to the synergistic treatment effects observed [32]. According to our results, although statistically significant improvements in survival outcomes were not seen in the subgroup of patients without BRCA/HRR mutations, sensitivity analysis pointed towards potential survival benefits for this subgroup upon exclusion of the KEYLYNK-010 study. This finding may be attributed to the use of pembrolizumab in the KEYLYNK-010 study. Similar sensitivity analyses were also conducted for time to prostate-specific antigen radiographic progression (TTPP) and prostate-specific antigen response rate (PSARR), both of which showcased the narrowing impact of PD-1 blockade therapy on the conclusions regarding survival benefits.

It has been postulated that patients without HRR mutations may still derive potential benefits from PARP inhibition [33]. On the one hand, PARP inhibitors can attenuate the transcriptional activity of the androgen receptor (AR), thereby enhancing the inhibitory effects of ARi on AR pathways [34,35]. On the other hand, AR itself serves as a transcriptional factor that promotes DNA damage response and HRR by facilitating the accumulation of γH2AX and RAD51 foci [36,37,38]. Consequently, the obstruction of AR signaling in patients undergoing androgen deprivation therapy (ADT) compromises HRR and leads to compensatory PARP activity. Thus, the inhibition of AR becomes synthetically lethal when combined with PARP inhibition [39,40]. Additionally, other yet undiscovered mutation loci may induce sensitivity to PARP inhibition in prostate cancer [41], which may explain why some mCRPC patients lacking deleterious HRR mutations still respond to PARP inhibitors [17]. Therefore, further delineation and more detailed studies of the HRR-negative population are warranted, as are additional fundamental research endeavors to uncover new mechanisms and improve the identification of the patient population suitable for PARP inhibitors.

Our comprehensive meta-analysis unequivocally demonstrates the substantial advantages conferred by PARP inhibitors over standard-of-care treatment in the improvement of rPFS and OS among mCRPC patients with any HRR mutation, thereby underscoring the immense therapeutic potential of PARP inhibitors in a stratified manner based on HRR gene-mutation signatures. Nonetheless, while our overall findings in the subgroup of patients without BRCA/HRR mutations do not advocate for the routine application of PARP inhibitors, sensitivity analysis reminds us that further exploration necessitates more profound and comprehensive pre-clinical and clinical evidence. Furthermore, the concept of “patient-centered clinical trials” has gained significant traction in recent times [42]. Despite the general responsiveness of BRCA/HRR-positive patients to PARP inhibitors, a subset of these patients fails to derive a survival benefit. The reasons for this subset of patients warrant in-depth subgroup analyses. Collectively, these findings underscore the necessity and significance of molecular testing in guiding patient management and emphasize the importance of establishing treatment frameworks that incorporate precisely targeted therapies for mCRPC patients.

However, it is important to acknowledge a concurrent elevation in the incidence of treatment-emergent adverse events. Regarding safety, the rates of overall adverse events (AEs), grade ≥ 3 AEs, and serious AEs were all higher in the PARP inhibitor treatment arm as compared to the standard-of-care treatment arm. The most frequently observed adverse effects encompassed fatigue, anemia, hypertension, thrombocytopenia, nausea, neutropenia, and others, mirroring the occurrences reported in previous studies involving other solid tumors [6,8]. Generally, these side effects can be effectively managed through supportive measures such as transfusion of blood components and growth factor therapy, as well as dose reduction and interruption when necessary. Notably, the incidences of treatment-emergent hypertension were similar irrespective of the utilization of PARP inhibitors, thereby offering an alternative treatment option for individuals who cannot tolerate the elevated blood pressure associated with ARi.

## 5. Conclusions

This meta-analysis presents compelling and robust data indicating the favorable clinical efficacy and tolerability of PARP inhibitor (PARPi) treatment, both as a monotherapy and in combination therapy, for the management of refractory metastatic castration-resistant prostate cancer (mCRPC) characterized by BRCA/HRR mutations. To further enhance therapy selection and optimize treatment outcomes, it is imperative to dedicate resources to comprehensively investigate and comprehend predictive markers and signatures associated with treatment response and resistance to PARP inhibitors. This endeavor will enable the development of personalized treatment approaches tailored to individual patients, maximizing therapeutic benefit.

## Figures and Tables

**Table 1:** Characteristics of the studies included.

Study (Year)	Clinical Trials Number	RCT Phase	PARP Inhibitors	Treatment Arm	Control Arm	Enrollment Time	Inclusion Criteria	Exclusion Criteria	HRR Gene Alteration Status Criteria	Median Treatment Duration Months (Range)	Primary Endpoints
Agarwal et al., 2023 [17]	NCT03395197	Ⅲ	Talazoparib	Talazoparib 0.5 mg QD + enzalutamide 160 mg QD	Placebo + enzalutamide 160 mg QD	7 January 2019 to 17 September 2020	Patients with mCRPC who were receiving ongoing androgen deprivation therapy; serum testosterone ≤ 50 ng/dL; ECOG performance status ≤ 1; Life expectancy ≥ 12 months	Any prior systemic cancer treatment initiated in the non-metastatic CRPC or mCRPC disease state; Prior treatment with second-generation androgen receptor inhibitors, a PARP inhibitor, cyclophosphamide, or mitoxantrone for prostate cancer.	BRCA1, BRCA2, PALB2, ATM, ATR, CHEK2, FANCA, RAD51C, NBN, MLH1, MRE11A, CDK12	PARPi group: 19.8 months (IQR, 8.8–26.9) for talazoparib and 22.2 months (IQR, 9.9–28.1) for enzalutamide. Control group: 16.1 months (IQR, 6.5–25.0) for placebo and 16.6 months (IQR, 6.7–25.1) for enzalutamide.	rPFS
Chi et al., 2023 [18]	NCT03748641	Ⅲ	Niraparib	Niraparib 200 mg QD + abiraterone acetate 1000 mg QD + prednisone 5 mg BID	Placebo + abiraterone acetate 1000 mg QD + prednisone 5 mg BID	May 2019 to March 2021	Patients with mCRPC and an ECOG performance status of 0 to 1; Score of ≤3 on the Brief Pain Inventory-Short Form (BPI-SF) Question	Patients have received prior PARP inhibitors or systemic therapy; Patients have the evidence of progression by PSA who received 2 to 4 months of AAP; Presence of uncontrolled hypertension(persistent systolic blood pressure [BP] ≥ 160 mmHg or diastolic BP ≥ 100 mmHg).	ATM, BRCA1, BRCA2, BRIP1, CDK12, CHEK2, FANCA, HDAC2, PALB2	HRR + group: 13.8 months (range, 0–29.0) in the PARPi group and 12.1 months (range, 0–29.0) in the control group. HRR − group: not mentioned.	rPFS
Clarke et al., 2023 [21]	NCT03732820	Ⅲ	Olaparib	Olaparib 300 mg BID + abiraterone acetate 1000 mg QD + prednisone 5 mg BID	Placebo + abiraterone acetate 1000 mg QD + prednisone 5 mg BID	31 October 2018 to 12 October 2022	Patients with mCRPC and an ECOG performance status of 0 to 1; no prior exposure to abiraterone; serum testosterone < 50 ng/dL	Patients have received prior cytotoxic chemotherapy or new hormonal agents (NHAs) at metastatic castration-resistant prostate cancer (mCRPC) stage.	ATM, BRCA1, BRCA2, BARD1, BRIP1, CDK12, CHEK1, CHEK2, FANCL, PALB2, RAD51B, RAD51C, RAD51D, RAD54L	PARPi group: 17.5 months for olaparib and 18.2 months for abiraterone. Control group: 15.7 months for placebo and 15.7 months for abiraterone.	rPFS
Clarke et al., 2018 [11]/Fred Saad et al., 2022 [10]	NCT01972217	Ⅱ	Olaparib	Olaparib 300 mg BID + abiraterone acetate 1000 mg QD + prednisone 5 mg BID	Placebo + abiraterone acetate 1000 mg QD + prednisone 5 mg BID	25 November 2014 to 14 July 2015	Patients had mCRPC and an ECOG performance status of 0–2 with no deterioration observed in the 2 weeks before the study; Patients had to be candidates for abiraterone therapy and a life expectancy of 12 weeks or longer.	Patients received more than two previous lines of chemotherapy or had previous exposure to second-generation antihormonal drugs or any previous treatment with olaparib; Patients diagnosed with other malignancies up to 5 years before trial entry, and those with any evidence of severe or uncontrolled systemic diseases.	ATM, BARD1, BRCA1, BRCA2, BRIP1, CDK12, CHEK1, CHEK2, FANCL, PALB2, PPP2R2A, RAD51B, RAD51C, RAD51D, RAD54L	PARPi group: 309 days (IQR, 145–457) for olaparib and 338 days (IQR, 169–588) for abiraterone. Control group: 253 days (IQR, 113–421) for placebo and 253 days (IQR, 130–429) for abiraterone.	rPFS
Antonarakis et al., 2023 [19]	NCT03834519	Ⅲ	Olaparib	Olaparib 300 mg BID + pembrolizumab 200 mg Q21D for up to 35 cycles	Abiraterone acetate 1000 mg QD + prednisone 10 mg BID or enzalutamide 160 mg QD	30 May 2019 to 16 July 2021	Patients with mCRPC and an ECOG performance status of 0 to 1; serum testosterone <50 ng/dL;	Patients have a known additional malignancy that is progressing or has required active treatment in the last 3 years; Patients have uncontrolled hypertension as indicated by systolic BP > 170 mm Hg or diastolic BP > 105 mm Hg.	BRCA1, BRCA2, ATM, BARD1, BRIP1, CDK12, CHEK1, CHEK2, FANCL, PALB2, PPP2R2A, RAD51B, RAD51C, RAD51D, RAD54L	5.0 months (range, 0.2–28.9) in the PARPi group and 4.1 months (range, 0.4–28.8) in the control group.	rPFS, OS
Fizazi et al., 2023 [20]	NCT02975934	Ⅲ	Rucaparib	Rucaparib 600 mg BID	Docetaxel or abiraterone acetate or enzalutamide	8 February 2017 to 2 February 2022	Patients with mCRPC; Patients had a history of disease progression after treatment with one previous second-generation androgen receptor pathway inhibitor.	Patients have received prior treatment with any PARPi or chemotherapy.	BRCA1, BRCA2, ATM	8.3 months (range, 0.2–46.0) in the PARPi group and 5.1 months (range, 0.3–30.4) in the control group.	rPFS
Hussain et al., 2018 [12]	NCT01576172	Ⅱ	Veliparib	Veliparib 300 mg BID + abiraterone acetate 1000 mg QD + prednisone 5 mg BID	Abiraterone acetate 1000 mg QD + prednisone 5 mg BID	May 2012 to December 2015	Patients had mCRPC with ECOG performance status of 0 to 2; testosterone < 50 ng/dL; normal organ function; no prior exposure to abiraterone acetate plus prednisone, and up to two prior chemotherapy regimens.	Patients have received chemotherapy, radiotherapy, or oral antifungal agents within 3 weeks prior to entering the study; brain metastases.	NA	NA	PSA RR
Thiery-Vuillemin et al., 2022 [16]/De Bono et al., 2020 [13]/Hussain et al., 2020 [14]/Roubaud et al., 2022 [15]	NCT02987543	Ⅲ	Olaparib	Olaparib 300 mg BID	Enzalutamide 160 mg QD or abiraterone 1000 mg QD + prednisone 5 mg BID	6 February 2017 to 4 June 2019	Patients with confirmed mCRPC whose disease had progressed after receiving a previous next-generation hormonal drug.	Any previous treatment with PARPi; previous treatment with DNAdamaging cytotoxic chemotherapy; other malignancies within the past 5 years.	BRCA1, BRCA2, ATM, BRIP1, BARD1, CDK12, CHEK1, CHEK2, FANCL, PALB2, PPP2R2A, RAD51B, RAD51C, RAD51D, RAD54L	7.6 months (range, 0.03–28.9) in the PARPi group and 3.9 months (range, 0.6–29.1) in the control group.	rPFS

**Table 2:** Characteristics of the patients included. * Patients with bone disease only.

Study (Year)	Number of Patient Groups		Median Age, Years (Range)		Baseline Serum PSA, µg/L		Gleason Score						Disease Site								ECOG Performance Status							
							<8		≥8		Unknown		Bone		Lymph Node		Visceral		Other Soft Tissue		0		1		2		Unknown	
	PARPi Group	Control Group	PARPi Group	Control Group	PARPi Group	Control Group	PARPi Group	Control Group	PARPi Group	Control Group	PARPi Group	Control Group	PARPi Group	Control Group	PARPi Group	Control Group	PARPi Group	Control Group	PARPi Group	Control Group	PARPi Group	Control Group	PARPi Group	Control Group	PARPi Group	Control Group	PARPi Group	Control Group
Agarwal et al., 2023 [17]	402	403	71 (IQR 66–76)	71 (IQR 65–76)	18.2 (IQR 6.9–59.4)	16.2 (IQR 6.4–53.4)	117 (29.1%)	113 (28.0%)	281 (69.9%)	283 (70.2%)	4 (1.0%)	7 (1.8%)	349 (86.8%)	342 (84.9%)	147 (36.6%)	167 (41.4%)	57 (14.2%)	77 (19.1%)	37 (9.2%)	33 (8.2%)	259 (64.4%)	271 (67.2%)	143 (35.6%)	132 (32.8%)	NA	NA	NA	NA
Chi et al., 2023 [18]	HRR + Patients: 212 HRR − Patients: 123	HRR + Patients: 211 HRR − Patients: 124	HRR + Patients: 69 (range 45–100) HRR − Patients: 72 (range 53–87)	HRR + Patients: 69 (range 43–88) HRR − Patients: 71 (range 52–85)	21.4 (range 0–4826.5)	17.4 (range 0.1–4400.0)	57 (27.0%)	62 (29.5%)	144 (68.2%)	142 (67.6%)	10 (4.7%)	6 (2.9%)	183 (86.3%)	170 (80.6%)	113 (53.3%)	95 (45.0%)	51 (24.1%)	39 (18.5%)	6 (2.8%)	15 (7.1%)	130 (61.3%)	146 (69.2%)	82 (38.7%)	65 (30.8%)	NA	NA	NA	NA
Clarke et al., 2023 [21]	399	397	69 (range 43–91)	70 (range 46–88)	17.90 (IQR 6.09–67.00)	16.81 (IQR 6.26–53.30)	121 (30.3%)	134 (33.7%)	265 (66.4%)	258 (65.0%)	13 (3.3%)	5 (1.3%)	349 (87.5%)	339 (85.4%)	215 (53.9%)	208 (52.4%)	55 (13.8%)	60 (15.1%)	NA	NA	286 (71.7%)	272 (68.5%)	112 (28.1%)	124 (31.2%)	NA	NA	1 (0.3%)	1 (0.3%)
Clarke et al., 2018 [11] /Fred Saad et al., 2022 [10]	71	71	70 (IQR 65–75)	67 (IQR 62–74)	86 (IQR 23–194)	47 (IQR21–199)	NA	NA	NA	NA	NA	NA	33 (46.5%) *	33 (46.5%) *	Soft-tissue disease (include visceral organs) only: 8 (11%) verus 11 (15%) Bone and soft-tissue disease: 30 (42%) verus 27 (38%)						34 (47.9%)	38 (53.5%)	36 (50.7%)	30 (42.3%)	1 (1.4%)	1 (1.4%)	0	2 (2.8%)
Antonarakis et al., 2023 [19]	529	264	71 (range 40–89)	69 (range 49–84)	52.9 (range 0.1–5000.0)	42.6 (range 0.1–4007.0)	147 (27.8%)	69 (26.1%)	367 (69.4%)	184 (69.7%)	15 (2.8%)	11 (4.2%)	221 (41.8%) *	112 (42.4%) *	Liver: 50 (9.5%) verus 34 (12.9%) Other: 258 (48.8%) verus 118 (44.7%)						255 (48.2%)	139 (52.7%)	272 (51.4%)	125 (47.3%)	2 (0.4%)	0	NA	NA
Fizazi et al., 2023 [20]	270	135	70 (range 45–90)	71 (range 47–92)	26.9 (range 0.1–1247)	28.8 (range 0–1039)	97 (35.9%)	39 (28.9%)	173 (64.1%)	96 (71.1%)	NA	NA	235 (87.0%)	114 (84.4%)	118 (43.7%)	60 (44.4%)	74 (27.4%)	46 (34.1%)	NA	NA	132 (48.9%)	68 (50.4%)	138 (51.1%)	67 (49.6%)	NA	NA	NA	NA
Hussain et al., 2018 [12]	79	74	68 (range 47–85)	69 (range 50–90)	36.4 (range 0.04–1074.4)	32.7 (range 0.8–1557.6)	NA	NA	NA	NA	NA	NA	68 (86.1%)	64 (86.5%)	53 (67.1%)	45 (60.8%)	21 (26.6%)	13 (17.6%)	16 (20.3%)	13 (17.6%)	50 (63.3%)	46 (62.2%)	28 (35.4%)	28 (37.8%)	1 (1.3%)	0	NA	NA
Thiery-Vuillemin et al., 2022 [16]/De Bono et al., 2020 [13]/Hussain et al., 2020 [14]/Roubaud et al., 2022 [15]	256	131	69 (IQR 63–74)	69 (IQR 64–73)	68.2 (IQR 24.1–294.4)	106.5 (IQR 37.2–326.6)	68 (26.6%)	32 (24.4%)	183 (71.5%)	95 (72.5%)	5 (1.9%)	4 (3.1%)	86 (33.6%) *	38 (29.0%) *	NA	NA	68 (26.6%)	44 (33.6%)	88 (34.4%)	41 (31.3%)	131 (51.2%)	55 (42.0%)	112 (43.7%)	71 (54.2%)	13 (5.1%)	4 (3.0%)	0	1 (0.8%)

## Supplementary Materials

The following supporting information can be downloaded at: https://www.mdpi.com/article/10.3390/medicina59122198/s1, Figure S1: Risk of bias summary of the included studies; Figure S2: Risk of bias graph of the included studies; Figure S3: Forest plots showing the sensitivity analysis of treatment efficacy in mCRPC patients by sequential omitting each included study, (A) rPFS based on intention-to-treat patients, (B) rPFS based on Non-HRR mutated patients, (C) rPFS based on BRCA mutated patients, (D) rPFS based on Non-BRCA mutated patients, (E) OS based on intention-to-treat patients, (F) OS based on BRCA mutated patients, (G) TFST based on intention-to-treat patients, (H) TTPP based on intention-to-treat patients, (I) PSA RR based on intention-to-treat patients, and (J) ORR based on intention-to-treat patients; Figure S4: Forest plots showing the association of treatment efficacy in mCRPC patients, (A) TFST, (B) TTPP, (C) PSA RR, and (D) ORR; Figure S5: Forest plots showing the association of adverse events with a high incidence rate in mCRPC patients, (A) All grades of anemia, (B) Grade ≥ 3 of anemia, (C) All grades of fatigue, (D) Grade ≥ 3 of fatigue, (E) All grades of hypertension, (F) Grade ≥ 3 of hypertension, (G) All grades of thrombocytopenia, (H) Grade ≥ 3 of thrombocytopenia, (I) All grades of neutropenia, (J) Grade ≥ 3 of neutropenia, (K) All grades of leukopenia, and (L) Grade ≥ 3 of leukopenia; Table S1: Meta-analysis of the primary efficacy and safety of PAPRi for the treatment of mCRPC.
