# Supplementary figures and images for: Poly (ADP-ribose) Polymerase Inhibitors in Patients with Metastatic Castration-Resistant Prostate Cancer: A Meta-Analysis of Randomized Controlled Trials
Source: Medicina (Kaunas). 2023 Dec 18;59(12):2198. doi: 10.3390/medicina59122198 (PMC10744677; doi:10.3390/medicina59122198)

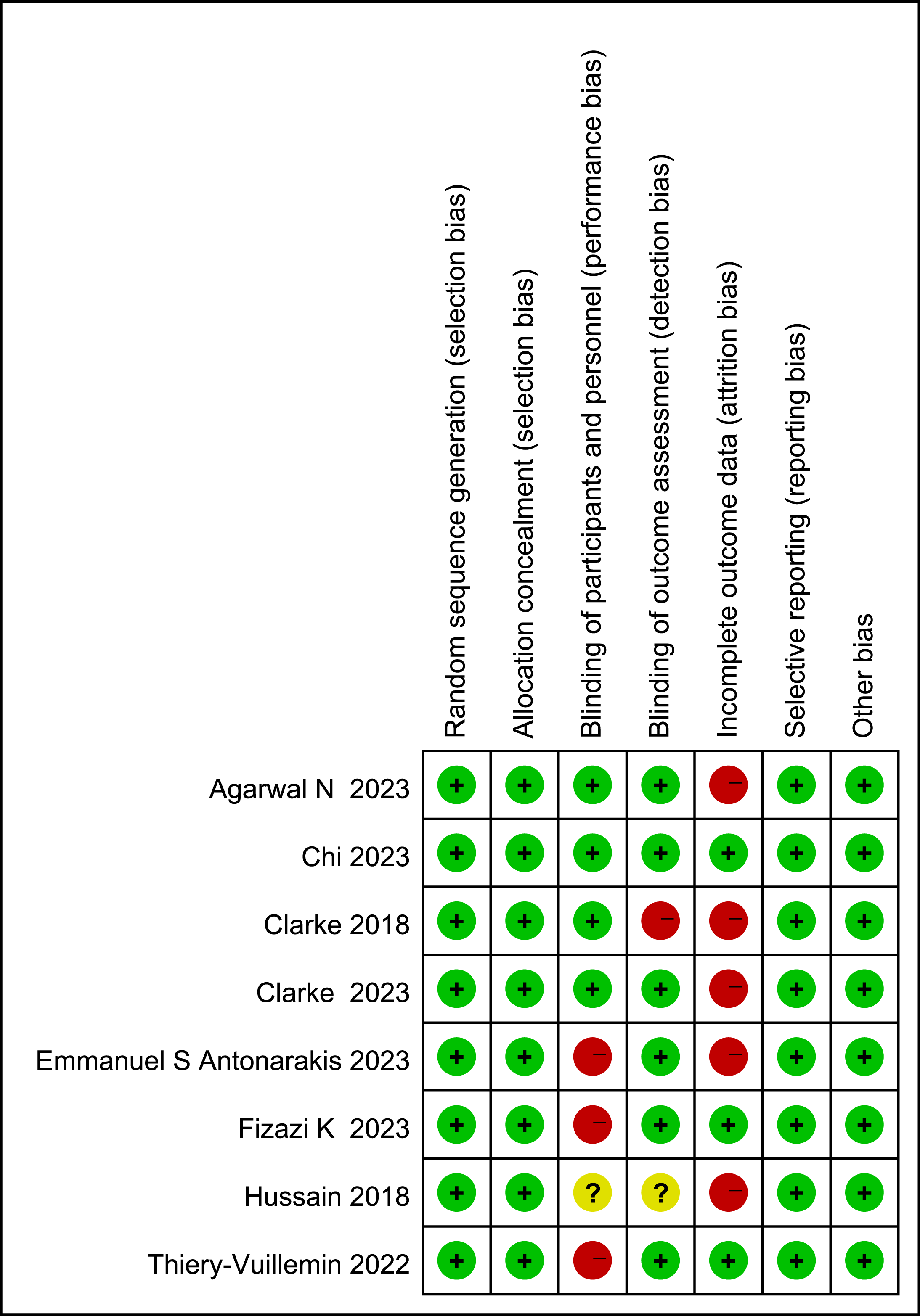

Supplement: Supplementary file 1 [file medicina-59-02198-s001.zip › Supplementary Figure S1 Risk of bias summary.tif]

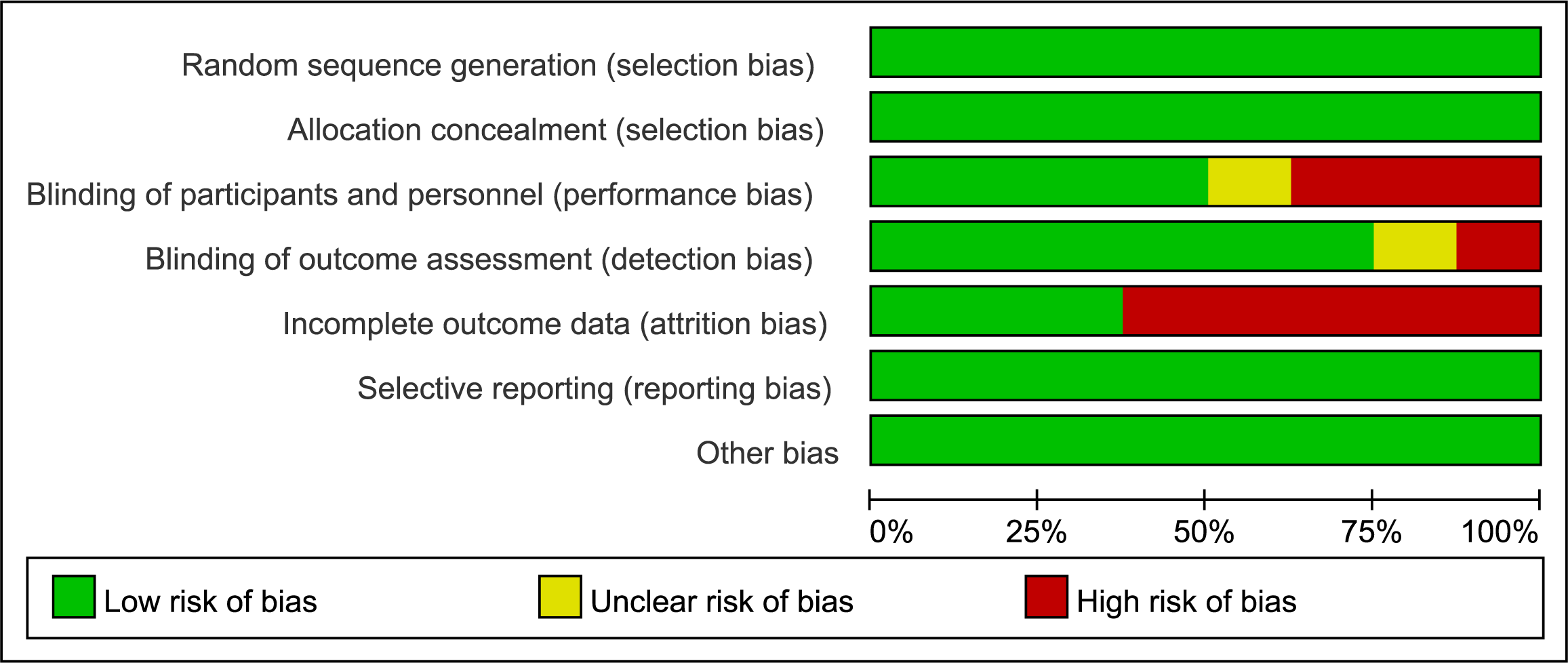

Supplement: Supplementary file 1 [file medicina-59-02198-s001.zip › Supplementary Figure S2 Risk of bias graph.tif]

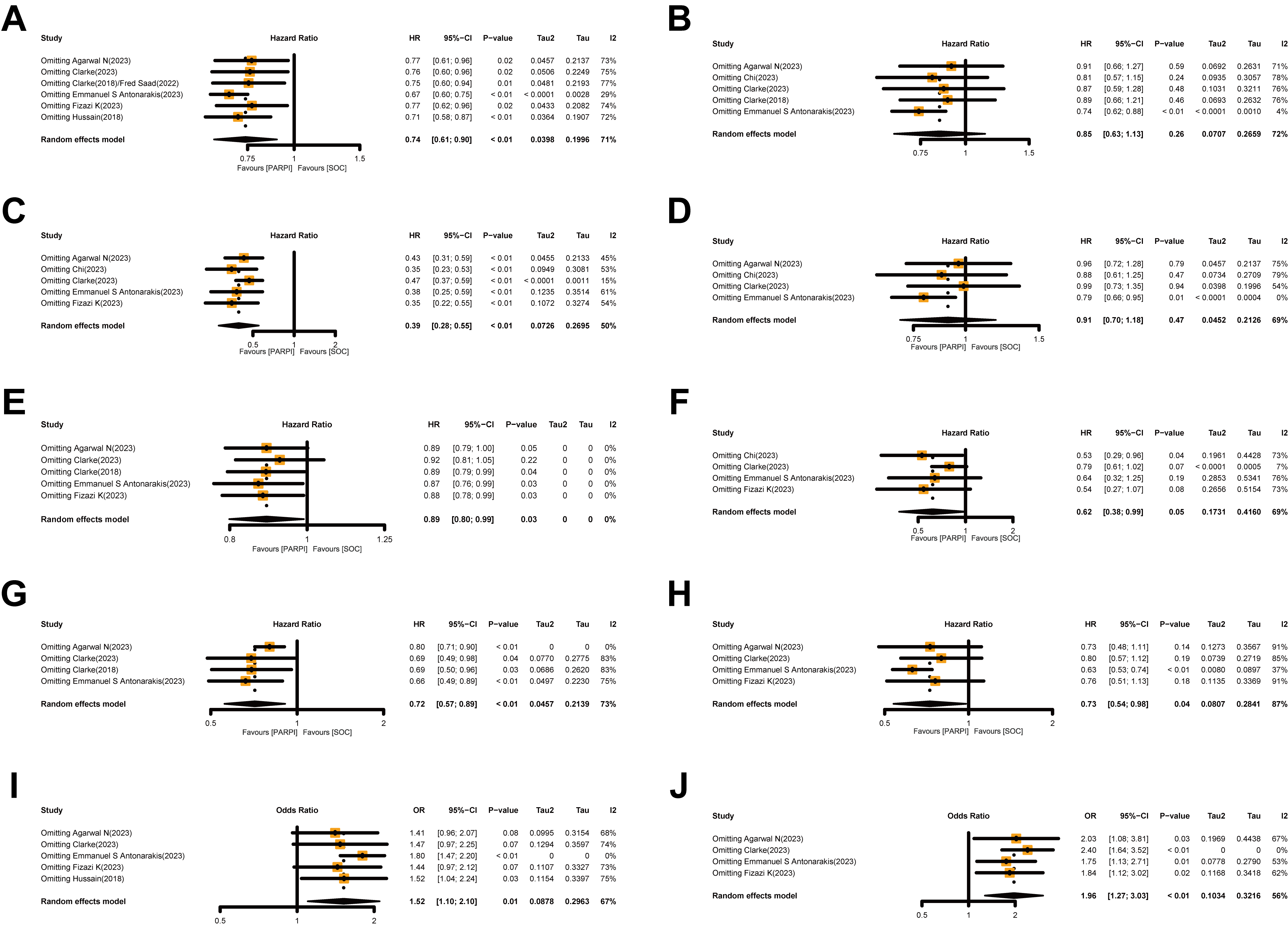

Supplement: Supplementary file 1 [file medicina-59-02198-s001.zip › Supplementary Figure S3 efficacy sensitive.tif]

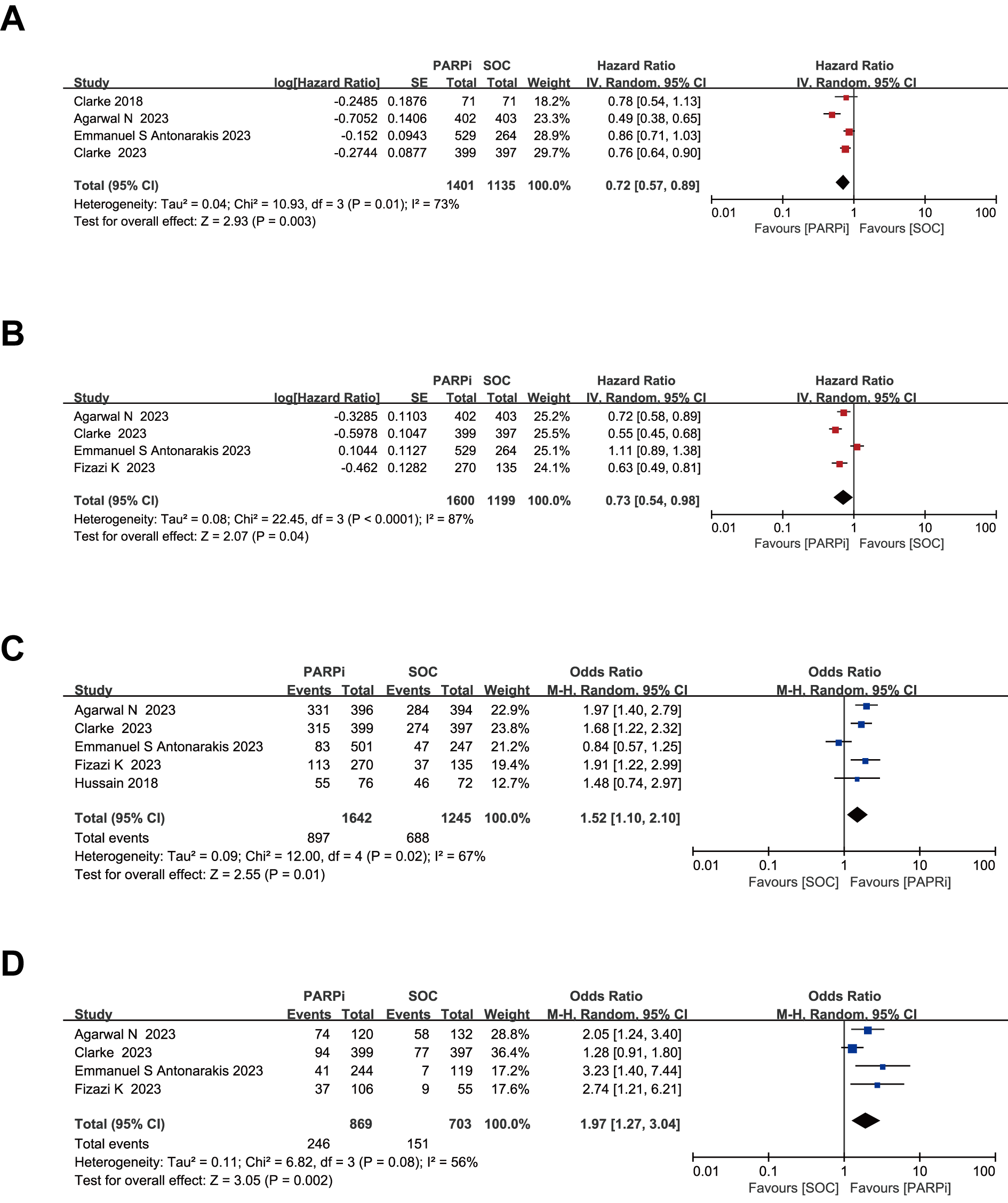

Supplement: Supplementary file 1 [file medicina-59-02198-s001.zip › Supplementary Figure S4 TPSTTTPPPSARRORR.tif]
